# Development of non-electrically controlled SalivaDirect LAMP (NEC-SD-LAMP), a new nonelectrical infectious disease testing method

**DOI:** 10.1038/s41598-023-38800-8

**Published:** 2023-07-21

**Authors:** Yusuke Kimura, Masashi Ikeuchi

**Affiliations:** 1 Department of Precision Biomedical Engineering, Institute of Biomaterials and Bioengineering, Tokyo Medical and Dental University, 2-3-10 Kandasurugadai, Chiyoda-ku, Tokyo, Japan; 2 Department of Advanced Functional Materials Research, Takasaki Advanced Radiation Research Institute, National Institutes for Quantum Science and Technology (QST), 1233 Watanuki-machi, Takasaki, Gunma Japan

**Keywords:** Infectious diseases, Viral infection, Gene expression analysis

## Abstract

In this study, non-electrically controlled SalivaDirect loop-mediated isothermal amplification (NEC-SD-LAMP), which can detect infections by amplifying viral DNA expression in saliva without using electrical control systems, was developed. By this method, only by adding water to the device, viral DNA was extracted from saliva using SalivaDirect, the extracted DNA was amplified via loop-mediated isothermal amplification (LAMP), and the results were visually confirmed. Melting palmitic acid maintained the optimal temperature for the LAMP reaction, as the temperature of palmitic acid is maintained at 62.9 °C, its melting point. By exploiting the proximity of the melting point to the optimal temperature for LAMP, LAMP can be performed without electricity. We detected several viruses in the saliva using this method. NEC-SD-LAMP could clearly distinguish 3 types of viral DNA, indicating the high specificity of this reaction. Furthermore, the viral concentration detection limit of the device was 2 copies per µL, indicating that it is possible to detect DNA viral infections in saliva even before the onset of viral infection.

## Introduction

Infectious diseases are major causes of death in developing countries^1,2^. One reason is the lack of diagnostic techniques for infectious agents. A more serious cause is the difficulty in accessing appropriate medical facilities^3^. This reduces the opportunities for testing, which is important for the diagnosis of viral infections. Early detection and treatment of viral infections are critical for complete cures and the prevention of their spread. In addition, infectious diseases, such as neglected tropical diseases are prevalent in many nonelectrified tropical areas, making it impossible to set up conventional testing devices requiring electrical control, so diagnostic techniques that require neither electrical infrastructure nor a stable voltage are important^4^. Therefore, to supply testing technology to developing countries, it is essential to establish methods that satisfy the following three criteria: (1) the entire process from testing to confirmation of results can be performed without using electrical control systems, (2) accurate detection of infectious diseases from an early stage, and (3) ease of use and portability that enables testing anywhere, such as in residential areas or outdoors, tropical or cold regions (point-of-care testing [POCT])^5^.

Several devices or methods can be used to perform infectious disease tests in nonelectrified environments. First, there are infection test kits based on antigen–antibody reactions, such as immunochromatography^6,7^. By adding samples to the kit, an infectious disease diagnostic test can be performed quickly, and the results can be observed visually or using a smartphone. Although these devices can easily perform tests, detecting infections in the early stages of a disease is difficult because of the protein detection methods employed by these devices. Nucleic acids can be efficiently amplified by Polymerase Chain Reaction (PCR) or Loop-mediated isothermal amplification (LAMP) and are effective in detecting infection at an early stage.

LAMP is an isothermal gene amplification method in which the results can be visually observed^8–10^.  This method amplifies and detects the target gene by heating the sample to a constant temperature of 60–65 °C. LAMP uses four or six primers during amplification, which has high specificity to the target gene. When the target gene is amplified by LAMP, pyrophosphate ions are produced. The pyrophosphate ions capture the manganese ions contained in the calcein-manganese ion complex in LAMP sample, causing the calcein to luminescent. As a result, the presence or absence of the target gene can be visually observed by the change of color of the sample^11^. From the above, LAMP has high specificity and detection sensitivity, as well as real-time PCR and qRT-PCR^12,13^, which are the gold standard methods for viral detection. LAMP is an easy-to-use method for viral detection compared to PCR in that the reaction can be performed with simple temperature control and the results can be visually confirmed.

To perform LAMP in a nonelectrified field, disposable pocket warmers have been used^14–19^. The LAMP method can be easily performed by placing a sample on disposable pocket warmers, and the results can be confirmed visually, showing improved portability. However, the output temperature of this method is unstable because the reaction is conducted using heat from disposable pocket warmers, which are considerably affected by the surrounding environmental temperature, especially in cold or tropical regions. Furthermore, the extraction of the target gene from a sample was not considered, other extraction processes might be needed.

In this study, we developed a new practical method named “Non-Electrically Controlled SalivaDirect LAMP (NEC-SD-LAMP),” which can extract viral genes from saliva, amplify the extracted viral DNA, and confirm the results visually by only adding water. This method extracts viral DNA from saliva using the SalivaDirect and amplifies viral genes using the LAMP, and viral infection can be confirmed with the naked eye. In the SalivaDirect, viral proteins in the saliva are denatured and deactivated by adding proteinase K. Simultaneously, the DNase and RNase in the saliva are inactivated so that the viral genes in the sample can be stably preserved. After the reaction, the sample was heated at 95 °C for 5 min to inactivate proteinase K, and the reaction was stopped and extract viral DNA from saliva.^20^.

NEC-SD-LAMP uses an exothermic agent and palmitic acid to perform the SalivaDirect and LAMP reactions in a reaction device comprising an insulated container. Since the reaction was performed in an insulated container, it could be conducted without being affected by the surrounding environmental temperature. When water is added to the NEC-SD-LAMP device, the exothermic agent within the device reacts and heats to 95 °C. This process terminates the SalivaDirect reaction. The heat produced by the exothermic agent is also used to melt palmitic acid. During the transition of any substance from liquid to solid, its temperature is maintained at the melting point of the substance. Applying this property, the temperature of the melt palmitic acid was fixed at the melting point (62.9 °C) during the transition of palmitic acid from liquid to solid. LAMP is performed by exploiting the proximity of the melting point of palmitic acid to the optimal temperature of the LAMP reaction.

Based on these principles, we achieved heating at approximately 95 °C for more than 5 min, which is necessary for proteinase K inactivation, and at 60–65 °C for more than 1 h, which is necessary for LAMP. This device is small (20 × 16 × 14 cm) and can be fabricated for less than USD 20, and all components, except for the exothermic agent and sample tube with which the specimen comes into contact, can be reused. Thus, this device is inexpensive and portable and can be used as a POCT in developing countries, including cold or tropical regions. When we used this device to detect adenovirus DNA in saliva, we detected a viral concentration as low as 2 copies per µL. Furthermore, we were able to detect 3 species of viral DNAs specifically. These results indicate that the early diagnosis of infectious diseases is possible using this device.

## Results

### Overview of the NEC-SD-LAMP device

**Figure 1:**
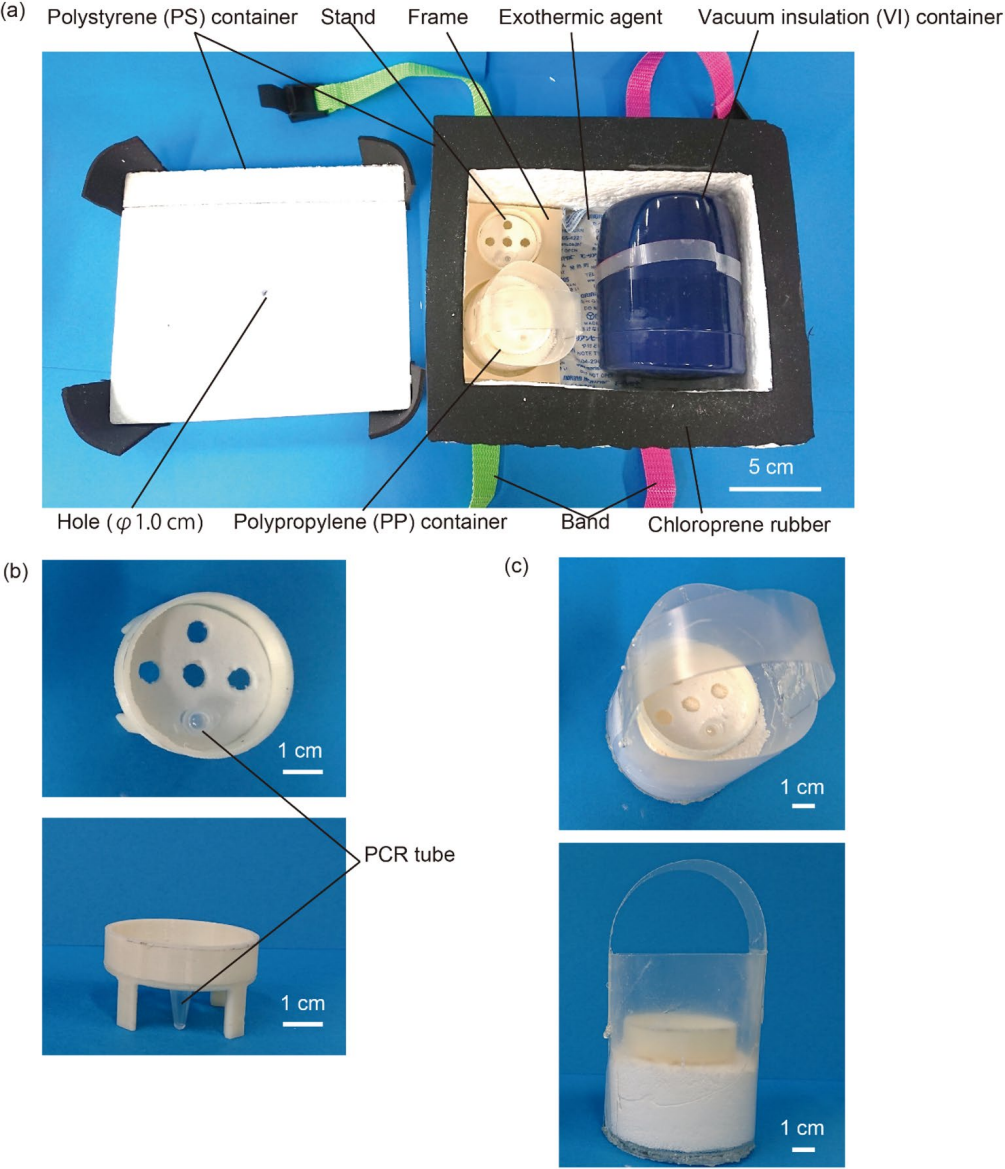
The developed NEC-SD-LAMP device. (**a**) All components of the NEC-SD-LAMP device. (**b**) Stand for the SalivaDirect. There are several holes in this component in which to place the PCR tubes. (**c**) Polypropylene (PP) container. The stands for the PCR tube and palmitic acid are mounted inside this container.

Figure 1a shows the fabricated NEC-SD-LAMP device. The device consisted of a stand on which to place a PCR tube with reagents when the SalivaDirect was performed, an exothermic agent, a polypropylene (PP) container containing palmitic acid, a vacuum-insulated (VI) container used during the LAMP, a frame to determine the position of these components, and a polystyrene (PS) container to hold all of these components. The stand and frame were fabricated using a 3D printer. The stand contained multiple holes for PCR tube placement, enabling simultaneous reactions of multiple samples (Fig. 1b). The stand is removable, and by using other stands, we could change the number of samples in an experiment or perform experiments on tubes with other volumes. The exothermic agents consisted of CaO and Al. The PP container was made of a 200-µm-thick PP film and fitted inside the VI container when performing the LAMP phase (Fig. 1c). The PS container was formed by combining 2.0-cm-thick polystyrene boards. The lid of the PS container has a hole 1.0 cm in diameter, which serves to release the hydrogen and water vapor generated during the SalivaDirect to the outside of the container. When performing the SalivaDirect reaction, the PS container was closed and secured using a band, and a 2.0-mm-thick chloroprene rubber stopper was mounted between the lid and the container to seal the container.

**Figure 2:**
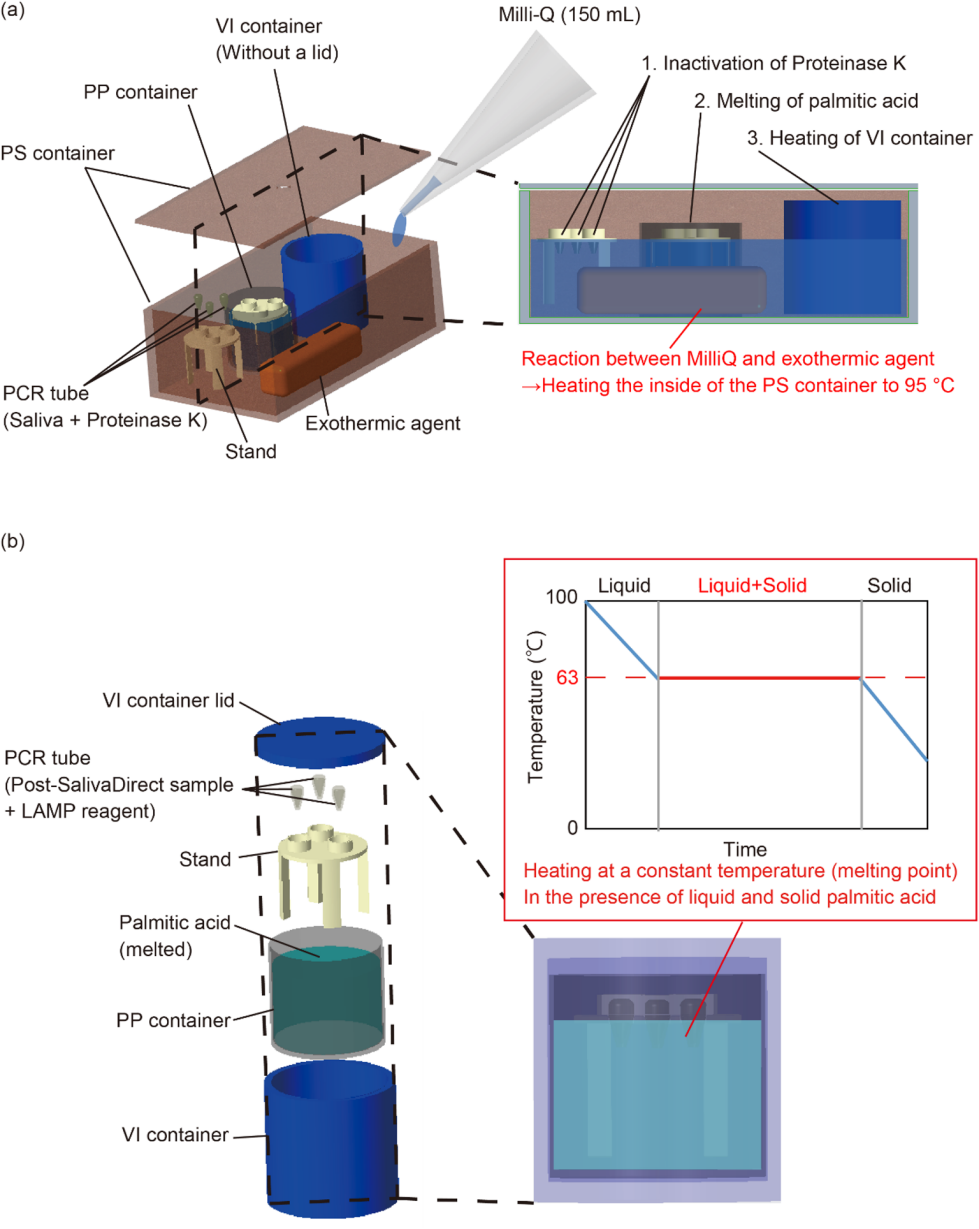
Mechanism of the NEC-SD-LAMP device. These Figures were created using PTC Creo parametric ver. 8.0.4.0. (https://www.ptc.com/ja/products/creo/parametric). (**a**) Operation of the SalivaDirect assay. In this method, the exothermic reaction between Milli-Q water and an exothermic agent is performed to stop the SalivaDirect by inactivating proteinase K. In this process, dissolving palmitic acid in the PP container and heating the inside of the vacuum-insulated (VI) container are also performed. (**b**) Operation of the LAMP assay. During the LAMP, the PP container is placed inside the VI container, and the PCR tube containing the LAMP sample is placed on the stand of the PP container. The reaction is carried out by applying the transition heat of palmitic acid as it transitions from liquid to solid.

Figure 2 shows the reaction process in the developed device. During the SalivaDirect reaction, the stand, exothermic agent, and PP and VI containers (without lids) were placed in a PS container (Fig. 2a). First, proteinase K was added to a saliva sample in a PCR tube, which was then placed on the stand for the SalivaDirect. After the reaction, Milli-Q water was added to the PS container, and the lid of the PS container was closed, initiating the following reactions between Milli-Q water and the exothermic agent:

1 $${\text{CaO}} + {\text{H}}_{{2}} {\text{O}} = {\text{CaOH}}_{{2}} + {15}.{\text{2 Kcal}}$$

2 $${\text{Al}} + {1}/{\text{2 Ca}}\left( {{\text{OH}}} \right)_{{2}} + {\text{ 2H}}_{{2}} {\text{O}} = {1}/{\text{2CaO}} \cdot {\text{Al}}_{{2}} {\text{O}}_{{3}} + { 3}/{\text{2H}}_{{2}} + {93.8}\;{\text{Kcal}}$$

The heat generated by this reaction was used to stop the SalivaDirect through proteinase K inactivation, melt the palmitic acid in the PP container, and heat the interior of the VI container.

LAMP was performed after the SalivaDirect. For the LAMP, the PP container was placed in the VI container (Fig. 2b). The post-SalivaDirect sample was then added to the LAMP reagent in a PCR tube and placed on a stand in the PP container. The lid of the VI container was closed, and the LAMP proceeded. The temperature in the PP container can be maintained at 62.9 °C, the melting point of palmitic acid, until all the palmitic acid in the PP container transitions from liquid to solid. Because the reaction is performed in a VI container, maintaining the temperature of palmitic acid and achieving a uniform reaction independent of the surrounding environmental temperature is possible. After the reaction, the solidified palmitic acid can be reused by removing the PP container from the VI container and reheating it. This dual-container structure allows repeated use without changing any device components except for the exothermic agent. This process enables the detection of viral infections without using any electrical control systems.

### Temperature verification

**Figure 3:**
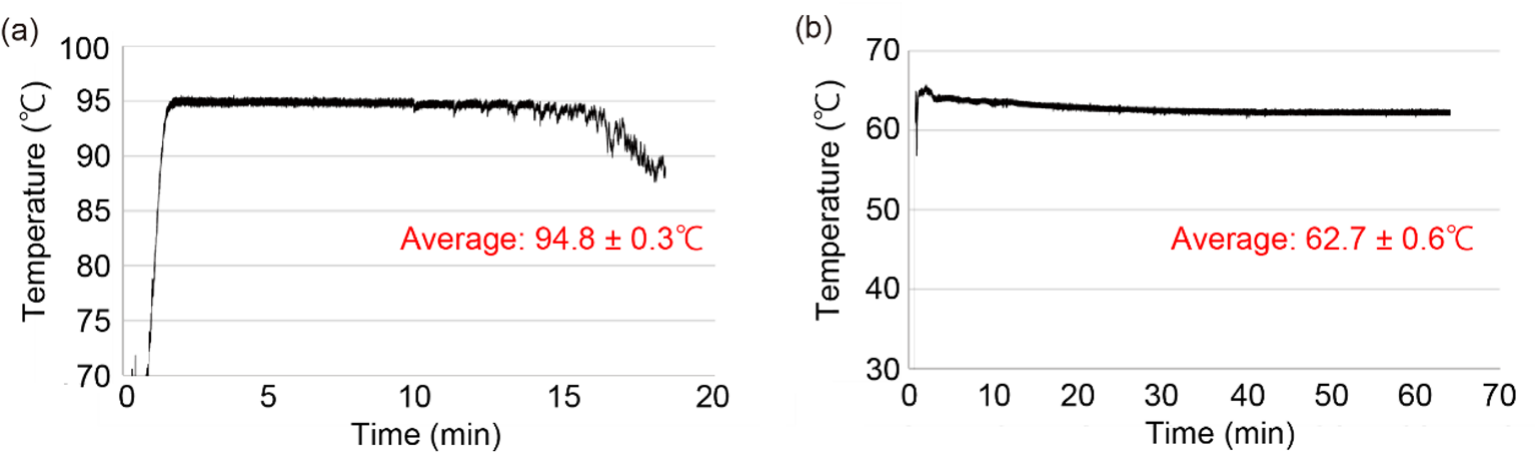
Temperature changes during each heating process. The graphs show the temperature in the container at each time. (**a**) Temperature change in the PS container during the exothermic reaction. (**b**) Temperature change of palmitic acid in the PP container during the LAMP.

Figure 3a shows the temperature change inside the PS container during the exothermic reaction. 150 mL of Milli-Q water was added to the PS container with the exothermic agent placed at the bottom. The temperature at each time point was calculated using the resistance of a thermistor placed on the side of the PS container. As shown in Fig. 3a, this reaction could heat the container to 94.8 °C for more than 5 min. This temperature and this reaction time are sufficient to inactivate proteinase K. We also confirmed that all the palmitic acid in the PP container could be melted by heating.

Figure 3b shows the temperature change of the palmitic acid in the PP container. This experiment verified the temperature change in melted palmitic acid after placing the PP container in the VI container after the exothermic reaction. The temperature at each time point was calculated using the resistance of a thermistor placed on the side of the PP container. As shown in Fig. 3b, the temperature of the palmitic acid in the PP container was successfully maintained at 62.7 °C for more than 1 h, which was sufficient to perform the LAMP.

### Viral DNA detection using NEC-SD-LAMP

**Figure 4:**
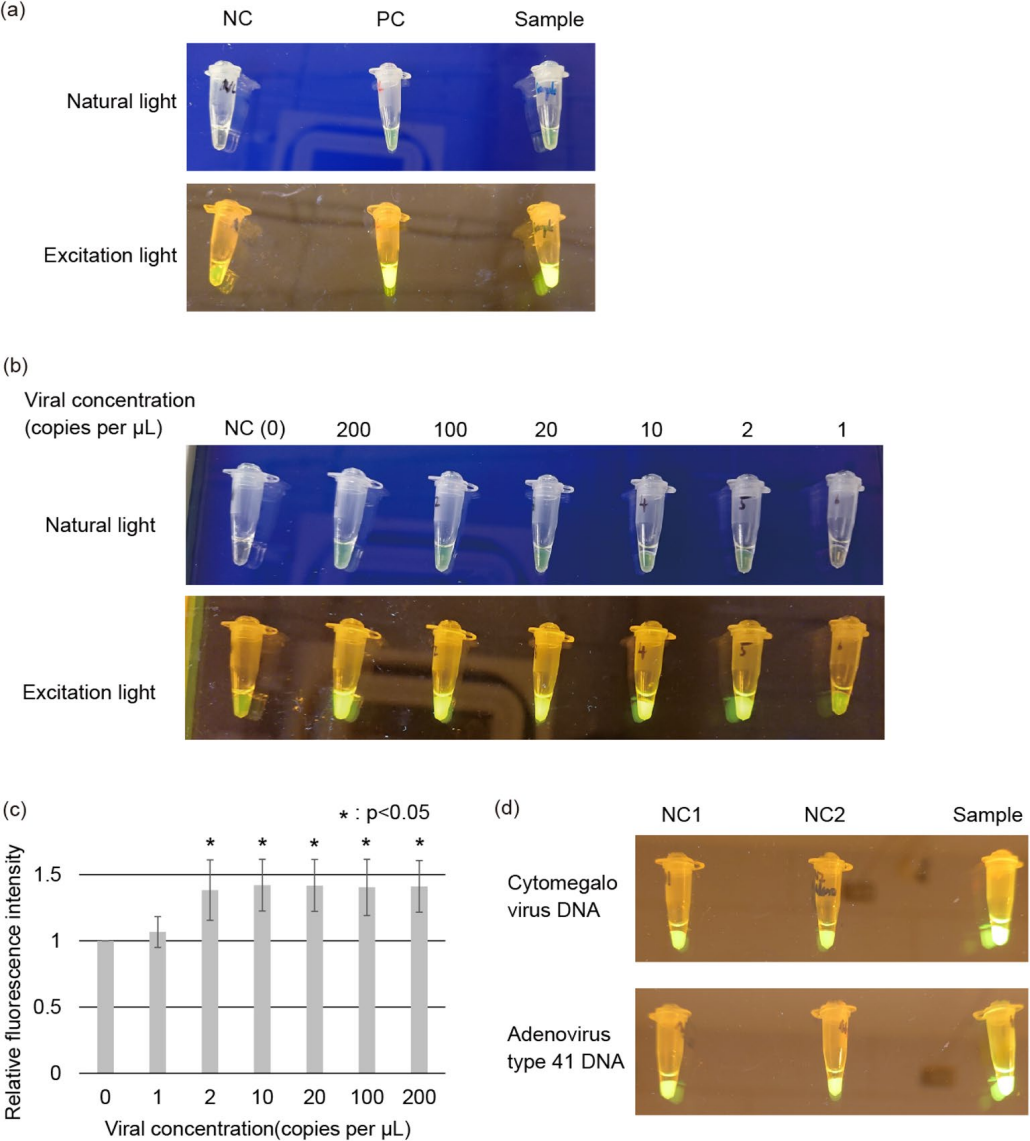
NEC-SD-LAMP experiment for detecting viral DNA. These photographs show the samples in the PCR tube after the reaction and were taken under natural or excitation light irradiation. (**a**) Functional comparison of NEC-SD-LAMP with a conventional system. “NC”, NEC-SD-LAMP was performed using saliva samples without adenovirus DNA. “PC”, SalivaDirect and LAMP reactions were performed using saliva samples containing adenovirus DNA in a conventional thermal cycler. “Sample”, NEC-SD-LAMP was performed using saliva samples containing adenovirus DNA. (**b**) Verification of the limit of detection of NEC-SD-LAMP. “NC”, NEC-SD-LAMP was performed using saliva samples without adenovirus DNA. (**c**) Analysis of the fluorescence intensity of samples containing each corresponding adenovirus DNA concentration using ImageJ software. The experiment was performed three times, and the relative fluorescence intensity of each experiment was calculated based on the fluorescence intensity of the NC sample. The mean and standard deviation are shown in the graph. (**d**) Verification of the specificity of NEC-SD-LAMP. “NC1”, NEC-SD-LAMP was performed using saliva samples without cytomegalovirus DNA or adenovirus type 41 DNA. “NC2”, NEC-SD-LAMP was performed using saliva samples containing cytomegalovirus DNA or adenovirus type 41 DNA, and adenoviral DNA-specific primers were used. “Sample”, NEC-SD-LAMP was performed using saliva samples containing cytomegalovirus DNA or adenovirus type 41 DNA, and each corresponding viral DNA detection primer was used.

Figure 4 shows the results of a test to detect viral DNA in saliva using the developed device. Figure 4a shows the PCR tube after NEC-SD-LAMP using saliva samples mixed with adenovirus DNA. Negative Control (NC) refers to NEC-SD-LAMP performed using saliva samples without adenovirus DNA, Positive Control (PC) refers to the SalivaDirect and LAMP performed using saliva samples containing adenovirus DNA from a conventional thermal cycler, and Sample refers to NEC-SD-LAMP performed using saliva samples containing adenovirus DNA. As shown in Fig. 4a, the color change of the sample solution was visually similar to that of the PC sample. It was possible to identify the samples under natural light.

To confirm that the SalivaDirect reaction was stopped by the exothermic reaction, NEC-SD-LAMP was performed on samples in which proteinase K was not inactivated. Gene amplification by LAMP reaction was not observed in samples even containing adenovirus DNA (Sup. Fig. 1). This result suggests that when proteinase K is not sufficiently inactivated, the enzyme in the LAMP reagent is inactivated, and the LAMP reaction does not proceed correctly. These results suggest that proteinase K can be sufficiently inactivated by an exothermic reaction.

The Supplementary Movie shows a video demonstration of using NEC-SD-LAMP. As shown in the Supplementary Movie, NEC-SD-LAMP can be used to extract genes from saliva, and the results can be observed and verified without any electrical control system.

### Using NEC-SD-LAMP in various temperature environments

The NEC-SD-LAMP device is designed for use in various areas, including cold or tropical regions. To verify whether a stable reaction could be performed in tropical regions, we set up the device in an incubator (50 °C) and performed NEC-SD-LAMP. Saliva-containing adenovirus DNA was used as the specimen, which was successfully detected in the incubator reaction at room temperature (Sup. Fig. 2). We also performed NEC-SD-LAMP in a freezer (− 20 °C) and succeeded in detecting adenovirus DNA in saliva (Sup. Fig. 3). These results indicate that the NEC-SD-LAMP system can perform stable reactions even in high- or low-temperature environments.

### Determining the detection limit of NEC-SD-LAMP

Figure 4b shows the detection limit verification results for determining the viral concentration using NEC-SD-LAMP. The PCR tube is shown after running NEC-SD-LAMP using saliva samples containing adenovirus DNA with final concentrations ranging from 0 to 200 copies per µL. As shown in Fig. 4b, samples containing more than 2 copies per µL of adenovirus DNA showed a change in solution color relative to the NC sample (0 copies per µL). Figure 4c shows the results of the fluorescence intensity analysis of the samples containing each virus concentration using ImageJ software. The experiment was performed three times, and the relative fluorescence intensity of each experiment was calculated based on the fluorescence intensity of the NC sample. The mean of each sample is shown in the graph. Error bars in the graph indicate the standard deviation. A t test was performed between the NC sample groups and samples containing each virus concentration. As shown in Fig. 4c, a significant increase in fluorescence intensity was observed in samples containing 2 or more copies per µL of adenovirus DNA. In contrast, a slight increase in fluorescence intensity was observed in samples containing 1 copy per µL. These results indicate that this method can visually detect samples containing more than 2 copies of viral DNA per µL. If fluorescence intensity analysis can be performed, it may be possible to detect virus concentrations as low as 1 copy per µL.

Sup. Fig. 4 shows the results of the detection limit for determining the viral concentration when the SalivaDirect and LAMP were performed using a conventional thermal cycler. When adenovirus DNA was detected with the conventional system, it was possible to visually confirm a color change of the solution of more than 1 copy per µL. Furthermore, under excitation light irradiation, more than 0.5 copies per µL could be detected, showing slightly higher detection sensitivity compared to the NEC-SD-LAMP.

We also tested the possibility of performing NEC-SD-LAMP in a large sample volume using a 1.5-mL tube, assuming the detection of saliva samples with extremely low viral concentrations. The NEC-SD-LAMP device can be equipped with a 1.5-mL tube by changing the stand (Sup. Fig. 4a,b), so we used a sample volume four times larger than that of a PCR tube. As a result, we were able to confirm a change in the solution color up to a virus concentration of 0.5 copies per µL (Sup. Fig. 4c), suggesting that NEC-SD-LAMP can be performed with only a change in the stand, even for large samples requiring a large amount of heat to perform the reaction. These results show that NEC-SD-LAMP could be used to detect viruses in saliva at very low concentrations.

### NEC-SD-LAMP with 3 species of virus DNA

Figure 4d shows the results of the NEC-SD-LAMP performed using 3 species of viral DNA. Saliva samples containing adenovirus type 41 or cytomegalovirus DNA were used. NEC-SD-LAMP was performed using primers specific for adenovirus, adenovirus type 41, and cytomegalovirus. As shown in Fig. 4d, changes in the color of the solution were observed when saliva samples contained the same virus species as the primers used. These results suggest that NEC-SD-LAMP can react specifically against the target virus.

## Discussion

By combining the SalivaDirect and LAMP, NEC-SD-LAMP can be used to test for viral infections in saliva samples without using electricity. Verification experiments were performed using 3 types of viral DNA. It is possible to detect viral DNA sequences not used in this study by accurately preparing the corresponding primer sequences.

Because the NEC-SD-LAMP system performs reactions in highly insulated PS and VI containers, it is not affected by environmental temperatures. The results of this study showed that the reactions could be carried out in ambient temperatures as low as − 20 °C, and as high as 50 °C. However, when conducting tests in tropical regions, it is necessary to bring not only the device but also reagents to the field. Conventional reagents are difficult to use because they must be stored below − 20 °C. Lyophilized reagents that can be stored at room temperature have since been developed^21^. Furthermore, these lyophilized reagents are already in practical use (DryADD, Nippon Gene Co., Ltd., Tokyo, Japan; https://www.nippongenematerial.com/dry_reagent/LAMP_Master_Mix.html). By combining the NEC-SD-LAMP system with lyophilized reagents enables field testing in tropical regions.

NEC-SD-LAMP could detect viral genes at a concentration as low as 2 copies per µL. In the 1.5-mL tube reaction, the viral concentration could be detected up to 0.5 copies per µL. One of the causes of the SARS-CoV-2 pandemic was the limited number of facilities available for testing, which prevented patients from being tested, resulting in the severity of symptoms or the spread of infection. The NEC-SD-LAMP system is not only highly sensitive and capable of detection from the early stages of infection but also is portable and can be performed with very simple operations. Therefore, it fulfills all the required factors for POCT and can help to solve the causes of the pandemic. In addition, the NEC-SD-LAMP device can be fabricated at low cost. Nucleic acid analyzers for LAMP can be provided at low cost to various research facilities and small laboratories by practical realization of NEC-SD-LAMP. The NEC-SD-LAMP system has the potential to cope with future outbreaks of infectious diseases.

Since the purpose of this study was to validate the function of NEC-SD-LAMP, only 1 set of primers was validated. If further validation of the optimal primer sequence is performed, the sensitivity of NEC-SD-LAMP may be improved. On the other hand, the results of performing the SalivaDirect and LAMP on the same sample using a conventional thermal cycler detected more than 0.5 copies per µL. In addition, since PCR, the gold standard of virus detection methods, has been reported to have the same detection sensitivity as the LAMP when used to detect viral DNA and RNA^16,22^, it is considered possible that PCR can detect more than 0.5 copies per µL. From the above, the detection sensitivity of NEC-SD-LAMP is lower than that of conventional methods. This is because NEC-SD-LAMP does not have precise temperature control compared to thermal cyclers. The heated palmitic acid immediately after the SalivaDirect reaction may be the temperature of the solution to rise above 65 °C. In such cases, the temperature exceeds the optimal reaction temperature for the LAMP, making it difficult to perform an accurate reaction. For more optimal reactions, it is necessary to clarify when the optimal temperature for each reaction is reached by applying a temperature-sensitive seal to the device for instance^16^.

The results of NEC-SD-LAMP showed that the detection of virus was much more sensitive when image J analysis was performed under excitation light irradiation. This indicates that NEC-SD-LAMP can improve the sensitivity by using an additional analysis system. We are currently developing the NEC-SD-LAMP microdevice, which combines the NEC-SD-LAMP system with our previously developed LAMP microdevice^23^. By developing the NEC-SD-LAMP microdevice with a direct connection system to smartphones and application for observing sample fluorescence intensity, which were developed when the LAMP microdevice was created, further cost reduction, portability, and higher sensitivity will be possible.

NEC-SD-LAMP is a system that can perform the amplification of the target nucleic acid from the saliva without electricity, aimed at detecting viruses at an early stage in any field setting. A method for performing LAMP in a simple procedure and without electrical control is the use of a chemo-exothermic reaction with a pocket warmer^14–19^. These are low-cost, easy-to-operate POCT devices that allow the LAMP method to be performed by placing the sample on the warmer. However, many of them have low detection sensitivity. One reason for this is that chemo-exothermic reactions are unstable in terms of temperature. Many papers do not describe or provide the validation of temperature changes per unit of time. However, since the detection of very low viral concentrations is possible in some cases^16^, early testing is possible if the optimal conditions, such as the proportion of components in the warmer and the surrounding environment temperature, can be established. However, the reaction in the pocket warmer has only been verified at room temperature, and it has also been shown that an accurate reaction cannot be carried out in a low-temperature environment^15^. Although improvements can be seen when reactions are run in insulated containers, there are no examples of successful LAMP methods at surrounding temperatures below 4 °C. NEC-SD-LAMP detected nucleic acids in a wide range of surrounding environments, from − 20 to 50 °C (Sup. Figs. 2 and 3). The ability to perform nucleic acid amplification in this temperature range is expected to provide nucleic acid amplification technology in almost all regions of the world. Furthermore, the ability to perform the extraction of viral DNA, which is not possible with LAMP using pocket warmers, makes the NEC-SD-LAMP an effective POCT system.

This study demonstrated the possibility of specific viral detection at the early stage of infection without using electricity. As the SalivaDirect method was originally developed for SARS-CoV-2 detection, NEC-SD-LAMP should be validated using RNA viruses. RNA degradation occurs when RNA is heated at high temperatures for a long time; in such cases, accurate detection cannot be performed. Therefore, we performed only validation experiments with DNA viruses, not RNA viruses. To accurately detect RNA viruses using NEC-SD-LAMP, the amount of exothermic agent and water used in the exothermic reaction should be investigated more precisely to optimize the heating temperature and time. All experiments in this study were performed with RT-LAMP reagents, and the same protocol can be used for RNA viruses as well as DNA viruses. Once the optimal conditions for RNA detection have been studied, NEC-SD-LAMP can be used for RNA viruses.

In order to accurately evaluate the specificity and sensitivity of NEC-SD-LAMP, it is necessary to perform a large number of limit detection concentrations for a large number of virus species, or to conduct validation experiments using saliva mixed with various virus species that are different from the target DNA virus. Furthermore, the specificity and sensitivity to actual viruses are unclear because evaluation tests using actual patient saliva samples have not been performed. Since the purpose of this study was to prove the gene detection capability of NEC-SD-LAMP, we have not been able to verify these tests. In the future, we plan to conduct research on these points in collaboration with university hospitals or other hospital facilities, and perform verification experiments using actual patient specimens.

The proposed system can perform LAMP by exploiting the fact that the melting point of palmitic acid is equal to the optimum temperature for LAMP. Palmitic acid is the richest saturated fatty acid in the human body^24^. Therefore, it is highly biocompatible and appropriate for POCT, and was used in this device. Other isothermal nucleic acid amplification methods include the smart amplification process (SmartAmp), recombinase polymerase amplification (RPA), and nucleic acid sequence-based amplification (NASBA)^25–28^. These methods can be performed in this system by using substances with melting points similar to the optimal temperature for each reaction or by using paraffin adjusted to an appropriate melting point. Therefore, this system could be applied to various types of reactions. However, because water vapor is generated during exothermic reactions, if a water-soluble substance is used for the isothermal amplification reaction, the reaction may not proceed accurately; therefore, it is preferable to use fat-soluble substances.

In the present study, we developed a novel method called NEC-SD-LAMP to detect infectious diseases by amplifying viral DNA from saliva without using an electrical control system. In this method, the entire reaction can be carried out by adding Milli-Q water to the device, which can be performed in nonelectrified environments, including the observation of the results. In addition, it is a small device that allows the reuse of its components, except for the exothermic agent, and can be fabricated for less than USD 20, making it possible to perform POCT at a low cost. Because the reaction is performed in an insulated container, it can be performed in developing countries, including cold or tropical regions, when lyophilized reagents are used. This device will make it possible to provide testing technology not only to developing countries but also to disaster areas where for example, large-scale power outages occur. If this device can be used to detect RNA as well as DNA, NEC-SD-LAMP is an innovative method that will allow the easy testing of viral infections at any site worldwide.

## Methods

### Fabrication of the NEC-SD-LAMP device

The PS container was fabricated by combining 2.0-cm-thick polystyrene boards. A 1.0-cm-diameter hole was created in the lid to direct the generated water vapor and hydrogen outside the container. A 2.0-mm-thick piece of chloroprene rubber was placed between the top of the container and lid, providing a seal between the lid and container. Two bands were placed around the PS container to fix the lid to the container.

The stand and frame were fabricated using a Mojo 3D printer (Stratasys, Rehovot, Israel) with an ABS resin (Mojo P430 QuickPack Print Engine, Stratasys).

The PP container was made of a 200-µm-thick PP film. The stand and 80 g of palmitic acid (Wako Pure Chemical Industries, Ltd., Osaka, Japan) were placed inside the container, and a handle made of PP film was placed on top of the PP container for easy operation. Palmitic acid (C_16_H_32_O_2_) is a type of saturated fatty acid with a melting point of 62.9 °C.

A commercially available exothermic agent (Morians Heat Pack size L; Morian Heat Pack Co. Ltd., Irima City, Japan; http://www.morians.co.jp/morians/structure.html) and a VI container (SR250; ASVEL, Yamatokoriyama-shi, Japan) were also used as components for the device. The main components of the exothermic agent consisted of calcium oxide and aluminum, and 45 g was used in this experiment.

### Reaction temperature measurement

Temperature measurements during exothermic reaction and LAMP reaction by palmitic acid were performed using a previously developed temperature control system^29^. Each temperature in the system was determined by measuring the resistance of a thermistor (104 JT; SEMITEC, Tokyo, Japan) at 50 Hz and performing calculations on a laptop. The resistance of the thermistor was input to a laptop using an analog I/O PC card (ADA16-8/2(CB)L; CONTEC Co., Ltd., Osaka, Japan), and the temperature calculation program was created using LabVIEW ver8.2 (NI, Austin, TX, USA). During the exothermic reaction, a thermistor was placed on the side of the PS container. During the LAMP reaction, the thermistor was placed on the side of the PP container after storage in the VI container to place the thermistor in contact with the melted palmitic acid. The averages and standard deviations of the measured temperatures from 3–13 to 3–63 min after commencing the measurements during the exothermic and LAMP reactions were calculated.

### NEC-SD-LAMP verification experiment

The following experiments were conducted to verify the functionality of the NEC-SD-LAMP system. All saliva samples were prepared by the first author by taking his saliva and mixing it with the adenovirus DNA. All viral DNA used in this experiment was purchased from Vircell S.L. This product is freeze-dried DNA isolated and extracted from adenovirus. 50 µL of saliva was mixed with AMPLIRUN ADENOVIRUS DNA CONTROL (MBC001; Vircell S.L., Grenada, Spain) to a final concentration of 100 copies per µL and added to a PCR tube. The SalivaDirect reaction was performed by mixing MagMAX™ Viral/Pathogen Proteinase K (Thermo Fisher Scientific, Waltham, MA, USA) with a saliva sample to a final concentration of 50 µg per µL and incubated at room temperature for 5 min. After the reaction, 30 µL of mineral oil (M-5904; Sigma‒Aldrich, St Louis, MO, USA) was added to prevent sample evaporation, and the PCR tube was placed on a stand. An exothermic agent, a VI container without a lid, a PP container, and a stand with a PCR tube were placed inside the PS container. 150 mL of Milli-Q water was added to react with the exothermic agent and heat the inside of the PS container to approximately 95 °C for 5 min to inactivate proteinase K. It was confirmed that all the palmitic acid in the PP container melted, PP container was placed in a VI container.

LAMP reagents were prepared by mixing a 2 × reaction mix, enzyme mix, distilled water, fluorescence detection reagent (Eiken Chemical Co., Ltd., Tokyo, Japan), and six primers (FI, BI, F3, B3, Loop F, and Loop B; 001–00890; LAMP Primer Design and Synthesis Service; Nippon Gene, Tokyo, Japan), according to the RT-LAMP kit protocol (Eiken Chemical Co., Ltd.). Table 1 shows the primer sequences used to detect adenovirus DNA. These primers were designed the sequences and synthesized by LAMP Primer Design and Synthesis Service (Nippon Gene, Tokyo, Japan). In this study, 1 set of primer sequences was validated for each virus species. The final concentration of each primer was adjusted to 2 µM (PI and BI primers), 0.25 µM (F3 and B3 primers), or 1 µM (Loop F and Loop B primers). 20 µL of the prepared LAMP reagent and 5 µL of the postSalivaDirect sample were added to a PCR tube (final adenovirus DNA concentration: 20 copies per µL). After adding 20 µL of mineral oil to prevent evaporation, the PCR tube was placed on a stand in a PP container. LAMP was then performed by closing the lid of the VI container and placing it on a laboratory table for 1 h. After the reaction, the PCR tube was removed from the stand, the color of the solution was observed under natural or excitation light using a SmartBlue Transilluminator (Greiner Bio-One, Frickenhausen, Germany), and each observation was captured using a camera (SO-03j; Sony Corporation, Tokyo, Japan).

**Table 1:** Primer sequences used in this study.

Target virus	Primer	Sequence
Adenovirus	FI	5'-GCCTCCTTCGTGCGTGATGATATGGTTGGACGCTGG-3'
	BI	5'-AGTCGCGCAGCTTGTTGAATCAAGGAAACCCTGGACTA-3'
	F3	5'-GAAGACGATCTGCCTGAAG-3'
	B3	5'-AGGGACAGGATAAGTATGACAT-3'
	LoopF	5'-CGGTAGGTCTTACAGACGC-3'
	LoopB	5'-GGTGACCTGCACGTCTAG-3'
Adenovirus type 41	FI	5'-GGTCTTACTGGTGCCGTGGCGGAAGGAGAGTGTGAGT-3'
	BI	5'-GCAGAGTTTCCTGTAGACGACGGTCGGATCCTCCTCAAGTA-3'
	F3	5'-TTGTGCTTACTAGGTCCGA-3'
	B3	5'-GGCGCTTTAAGGACAAGT-3'
	LoopF	5'-AACAAGGCAGCTCAGTCTC-3'
	LoopB	5'-CCAGCTGTTGATTGCATAGAAG-3'
Cytomegalo virus	FI	5'-TCTGAACGTGCTGGTGATTACCTGTAGGTATCGCTCGGAG-3'
	BI	5'-TGCCCACGATGCACATCAAACACCTTAAACAGCACCG-3'
	F3	5'-ACAATCAACAGATCGGCTAC-3'
	B3	5'-CTTAACGCAGGTGAGCAA-3'
	LoopF	5'-CATCCTGTACTACCGTCGTAAG-3'
	LoopB	5'-ACCGAGTGTACTCGAACAAC-3'

For comparison, the following reactions were performed.

As a negative control (NC), a saliva sample without AMPLIRUN ADENOVIRUS DNA CONTROL was used, and NEC-SD-LAMP was performed using the same reaction described above.

As a positive control (PC), a saliva sample containing the same concentration of AMPLIRUN ADENOVIRUS DNA CONTROL was prepared and heated at 95 °C for 5 min to stop the SalivaDirect reaction and at 63 °C for 1 h for the LAMP reaction using a T100 Thermal Cycler (Bio-Rad Laboratories, Hercules, CA, USA).

To verify the effect of proteinase K inactivation, we prepared a sample that was not heated by an exothermic agent after the SalivaDirect reaction. LAMP with palmitic acid was used in this study. The amount of sample and concentration of reagents used in each process were the same as those described above.

To verify the functionality of the NEC-SD-LAMP device in tropical regions, it was installed in an incubator (EO-450 V; AS ONE Corporation, Osaka, Japan) heated to 50 °C, and NEC-SD-LAMP was performed. The amount of sample and concentration of reagents used in each process were the same as those described above.

To verify the functionality of the NEC-SD-LAMP device in cold regions, it was installed in a freezer (SR-D40C; SANYO, Osaka, Japan) cooled to − 20 °C, and NEC-SD-LAMP was performed. The amount of sample and concentration of reagents used in each process were the same as those described above. Considering the effect of the temperature rise in the freezer due to the exothermic reaction, during the LAMP reaction phase used by the palmitic acid, the reaction was performed in a VI container set up in another freezer cooled to − 20 °C.

### Verification of the detection limit of the device

To verify the limit of detection of NEC-SD-LAMP, the following experiments were performed.

All saliva samples were prepared by the first author by taking his saliva and mixing it with the adenovirus DNA. All viral DNA used in this experiment was purchased from Vircell S.L. 50 µL of saliva was mixed with AMPLIRUN ADENOVIRUS DNA CONTROL to a final concentration of 1000 copies per µL and added to a PCR tube. The same procedure detailed in the “NEC-SD-LAMP Verification Experiment” section was used to perform the SalivaDirect and exothermic reactions.

The LAMP method with palmitic acid heating was performed using the same procedure as in the “NEC-SD-LAMP verification experiment,” after which the color of the solution was observed under natural or excitation light on a SmartBlue Transilluminator. For the LAMP, the postSalivaDirect reaction sample was diluted using Milli-Q water to final concentrations of 1, 2, 10, 20, 100, and 200 copies per µL of adenovirus DNA, and each observation was captured using a camera. These images were analyzed using ImageJ software (National Institutes of Health).

As a comparison experiment, the following reactions were performed.

As a negative control (NC), a saliva sample without AMPLIRUN ADENOVIRUS DNA CONTROL was used, and NEC-SD-LAMP was performed using the same reaction described above.

To confirm the limit detection concentration by the conventional method, a saliva samples containing the same concentration of AMPLIRUN ADENOVIRUS DNA CONTROL was prepared and heated at 95 °C for 5 min to stop the SalivaDirect reaction and at 63 °C for 1 h for the LAMP reaction using a T100 Thermal Cycler.

As a large-volume NEC-SD-LAMP experiment, NEC-SD-LAMP was performed in a 1.5-mL tube. The reagent concentration was the same as that described above, and the reaction was performed with a four-fold increase in the total solution volume. The final concentration of adenovirus DNA was adjusted to 0 (NC), 0.5, and 1 copy per µL.

### Verification of the specificity of the NEC-SD-LAMP reaction

The following experiments were performed to verify the specificity of NEC-SD-LAMP. All saliva samples were prepared by the first author by taking his saliva and mixing it with the adenovirus type 41 or cytomegalovirus DNA. All viral DNA used in this experiment was purchased from Vircell S.L. This product is freeze-dried DNA isolated and extracted from adenovirus type 41 or cytomegalovirus. 50 µL of saliva was mixed with AMPLIRUN ADENOVIRUS 41 DNA CONTROL (MBC114, Vircell) or AMPLIRUN CYTOMEGALOVIRUS DNA CONTROL (MBC016, Vircell) to a final concentration of 100 copies per µL and added to a PCR tube. The same procedure detailed in the section “NEC-SD-LAMP verification experiment” was used to perform the SalivaDirect and exothermic reactions. The LAMP method with palmitic acid heating was performed using the same procedure as in the “NEC-SD-LAMP verification experiment,” after which the color of the solution was observed under natural or excitation light on a SmartBlue Transilluminator. Table 1 shows the primer sequences used in this experiment. These primers were designed the sequences and synthesized by LAMP Primer Design and Synthesis Service (Nippon Gene, Tokyo, Japan).

As a comparison experiment, the following reactions were performed.

As negative control 1 (NC1), a saliva sample without viral DNA was used, and NEC-SD-LAMP was performed using the same reaction as described above.

A saliva sample containing each viral DNA was used as negative control 2 (NC2), and NEC-SD-LAMP was performed using an adenoviral DNA detection primer.

## Supplementary Information

Supplementary Movie 1.

Supplementary Figures.

## Data Availability

All data generated or analyzed during this study are included in this published article (and its Supplementary Information files).
