# Supplementary material for: Development of non-electrically controlled SalivaDirect LAMP (NEC-SD-LAMP), a new nonelectrical infectious disease testing method
Source: Sci Rep. 2023 Jul 21;13:11791. doi: 10.1038/s41598-023-38800-8 (PMC10362045; doi:10.1038/s41598-023-38800-8)
Supplement: Supplementary file 2 — Supplementary Figures. [file 41598_2023_38800_MOESM2_ESM.docx]

**Supplementary Information**

**Development of Non-Electrically Controlled SalivaDirect LAMP (NEC-SD-LAMP), a New Nonelectrical Infectious Disease Testing Method**

Yusuke Kimura^1, 2^, Masashi Ikeuchi^1*^

^1^Department of Precision Biomedical Engineering, Institute of Biomaterials and Bioengineering, Tokyo Medical and Dental University, 2-3-10 Kandasurugadai, Chiyoda-ku, Tokyo, Japan

^2^ Department of Advanced Functional Materials Research, Takasaki Advanced Radiation Research Institute, National Institutes for Quantum Science and Technology (QST), 1233 Watanuki-machi, Takasaki, Gunma, Japan

*Correspondence and requests for materials should be addressed to M.I. (email: ikeuchi.mech@tmd.ac.jp)

**
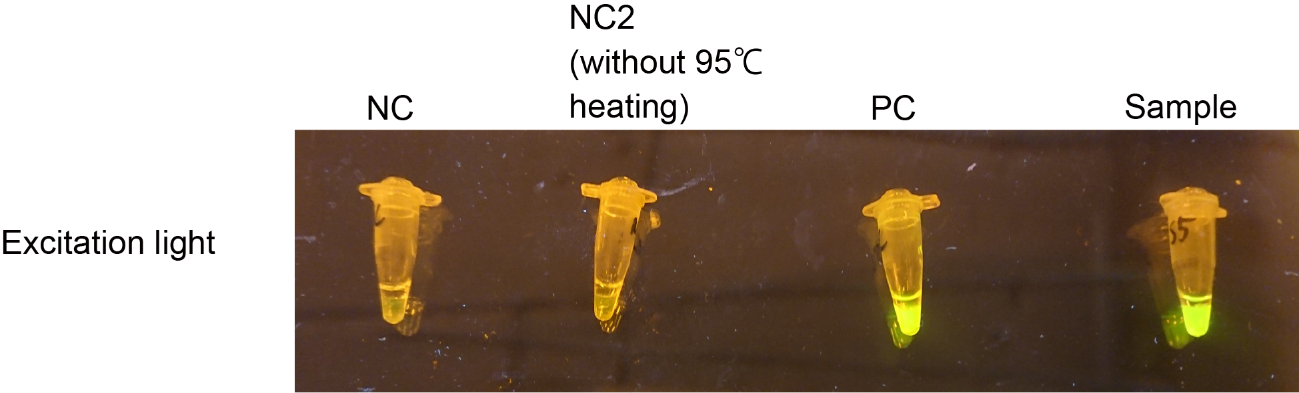
**

**Sup.Fig.1. Verification of the effect of proteinase K inactivation by the exothermic agent reaction**. The figure shows the samples in the PCR tube after reaction under excitation light illumination. “NC,” NEC-SD-LAMP was performed using saliva samples without adenovirus DNA. “NC2” used saliva samples containing adenovirus DNA, and the SalivaDirect was performed without inactivating proteinase K through the exothermic reaction. After the reaction, the LAMP was performed using palmitic acid. “PC,” SalivaDirect and LAMP were performed using saliva samples containing adenovirus DNA using a conventional thermal cycler. “Sample,” NEC-SD-LAMP was performed using saliva samples containing adenovirus DNA.


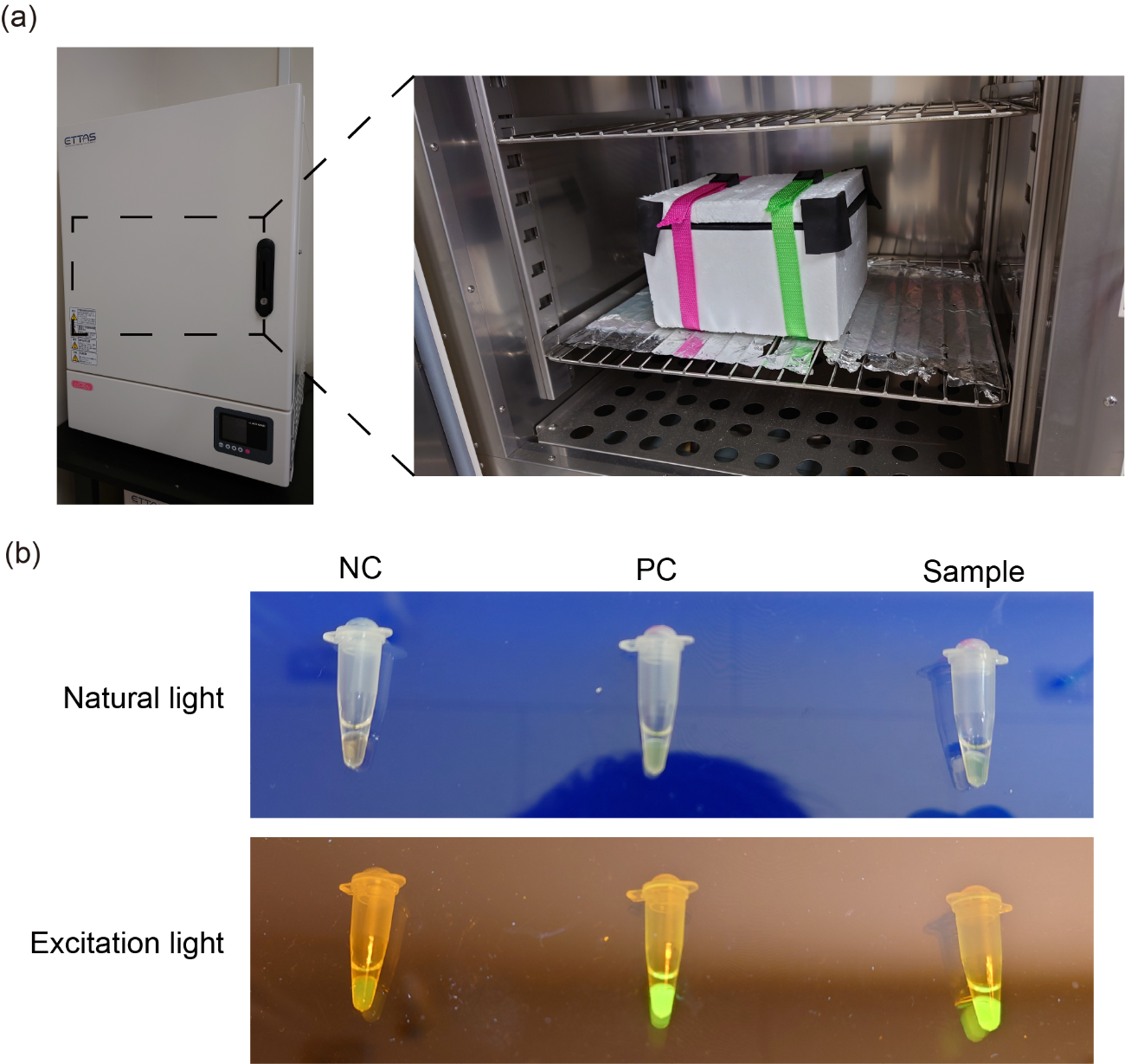


**Sup.Fig.2. NEC-SD-LAMP at 50 °C**. In this experiment, saliva samples containing adenovirus DNA (final concentration: 100 copies per µL) were used, and NEC-SD-LAMP was performed at the same volume, reagent concentration, and reaction time, as described in the method. (a) View of the NEC-SD-LAMP system during the reaction. All containers were placed in an incubator at 50 °C. (b) Reaction results: these figures show the samples in the PCR tubes after the reaction and were obtained under natural or excitation light irradiation. “NC,” NEC-SD-LAMP was performed using saliva samples without adenovirus DNA in a 50 °C incubator. “PC,” SalivaDirect and LAMP were performed using saliva samples containing adenovirus DNA in a conventional thermal cycler. “Sample,” NEC-SD-LAMP was performed using saliva samples containing adenovirus DNA in a 50 °C incubator.


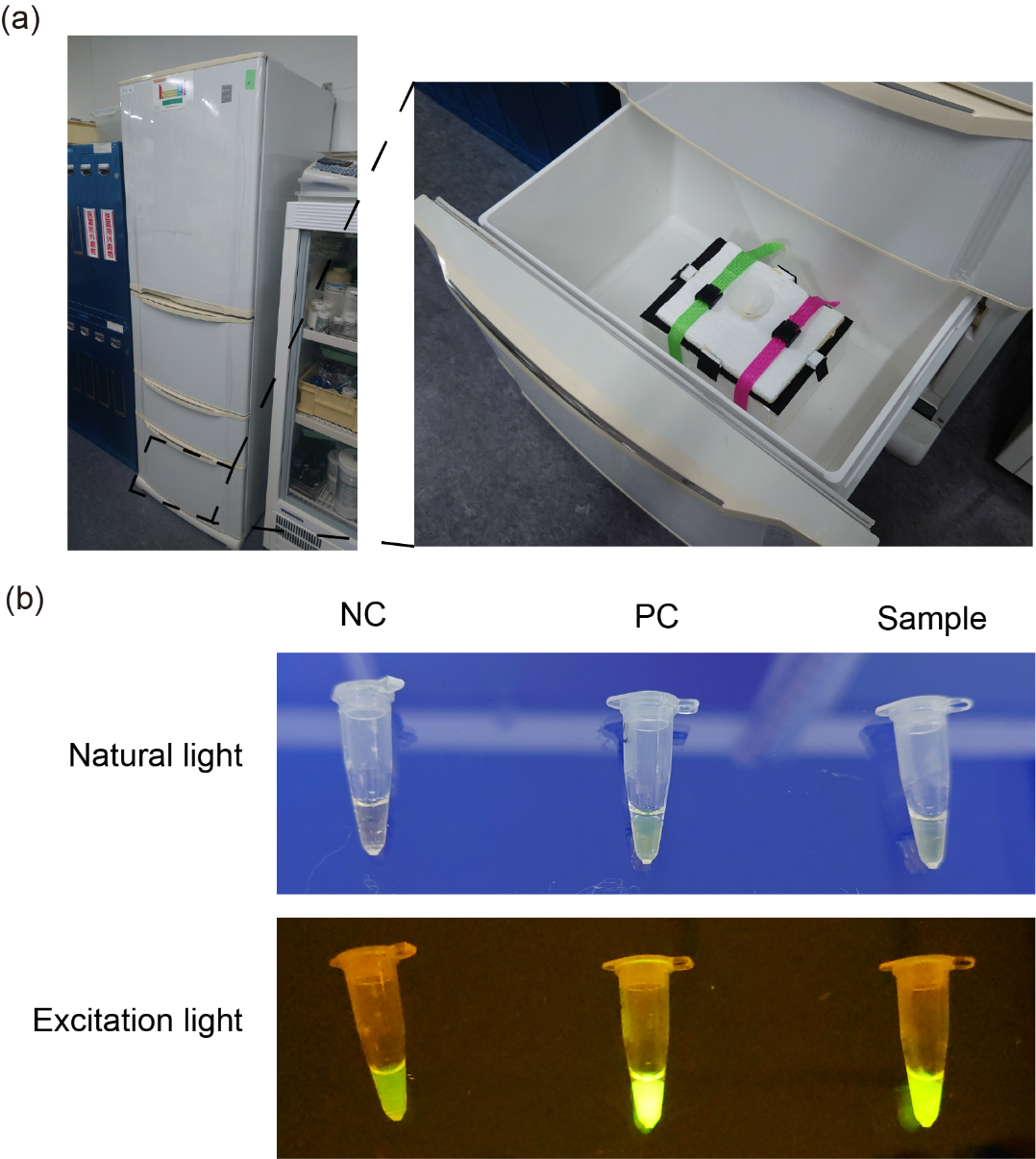


**Sup.Fig.3. NEC-SD-LAMP at -20 °C**. In this experiment, saliva samples containing adenovirus DNA (final concentration: 100 copies per µL) were used, and NEC-SD-LAMP was performed at the same volume, reagent concentration, and reaction time, as described in the method. (a) View of the NEC-SD-LAMP system during the reaction. All containers were placed in a freezer at -20 °C. Considering the effect of the temperature rise in the freezer due to the exothermic reaction, during the LAMP reaction phase used by the palmitic acid, the reaction was performed in a VI container set up in another freezer cooled to -20°C. (b) Reaction results: these figures show the samples in the PCR tubes after the reaction and were obtained under natural or excitation light irradiation. “NC,” NEC-SD-LAMP was performed using saliva samples without adenovirus DNA in a -20 °C freezer. “PC,” SalivaDirect and LAMP were performed using saliva samples containing adenovirus DNA in a conventional thermal cycler. “Sample,” NEC-SD-LAMP was performed using saliva samples containing adenovirus DNA in a -20 °C freezer.

**
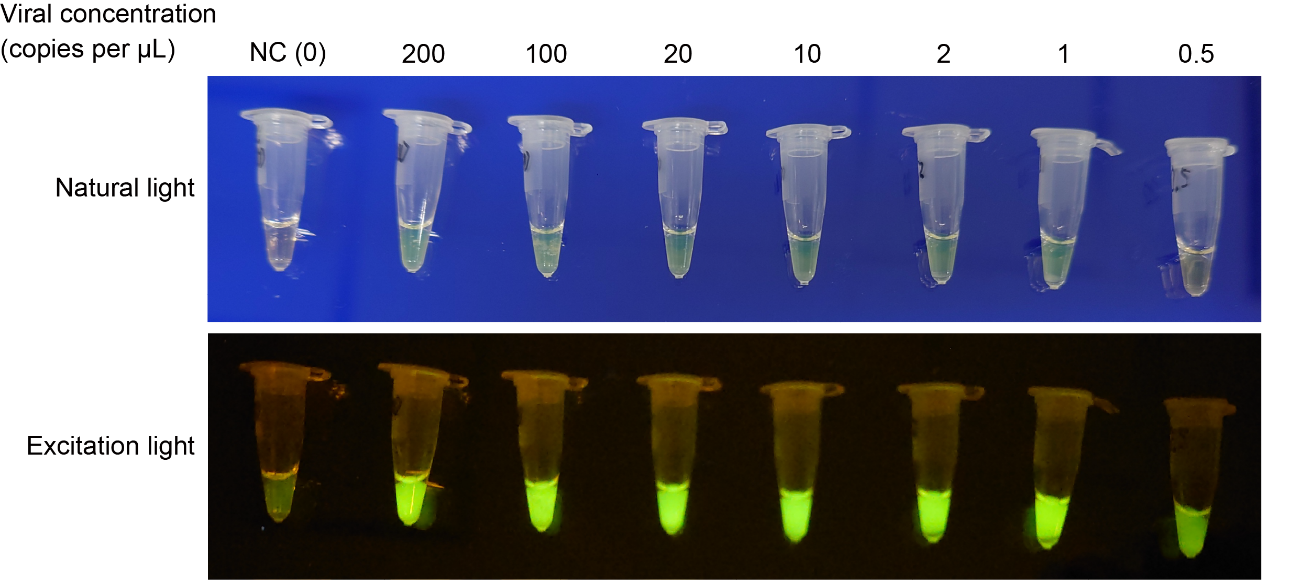
**

**Sup.Fig.4. Verification of the limit of detection of conventional thermal cycler.** These photographs show the samples in the PCR tube after the reaction performing SalivaDirect and LAMP at the T100 Thermal Cycler and were taken under natural or excitation light irradiation. “NC,” NEC-SD-LAMP was performed using saliva samples without adenovirus DNA.

**
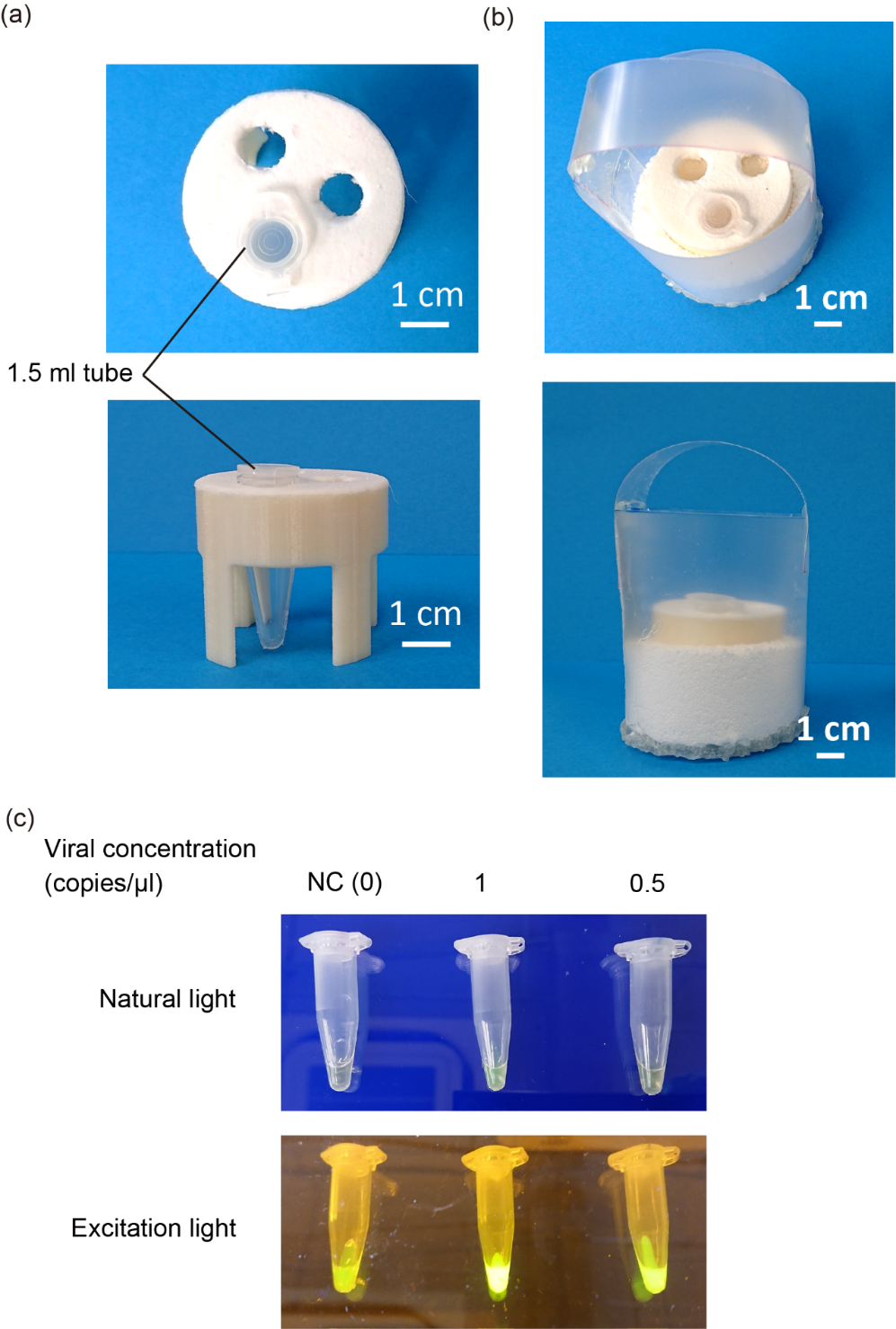
**

**Sup.Fig.5. NEC-SD-LAMP in a large volume using a 1.5-mL tube.** This experiment used saliva samples containing adenoviral DNA. NEC-SD-LAMP was performed as previously described. (a) Stand for the SalivaDirect assay. There are several holes on the stand to set the 1.5-mL tube. The stand is an attachment-type stand that can easily be replaced with a PCR tube stand. (b) PP container: the stand for the 1.5-mL tube and palmitic acid were mounted inside the container. (c) Reaction results: these figures show the sample in the 1.5-mL tube after the reaction and were obtained under natural or excitation light irradiation. “NC,” NEC-SD-LAMP was performed using saliva samples without adenovirus DNA.
